# Generalisation and specialisation in hoverfly (Syrphidae) grassland pollen transport networks revealed by DNA metabarcoding

**DOI:** 10.1111/1365-2656.12828

**Published:** 2018-04-16

**Authors:** Andrew Lucas, Owen Bodger, Berry J. Brosi, Col R. Ford, Dan W. Forman, Carolyn Greig, Matthew Hegarty, Penelope J. Neyland, Natasha de Vere

**Affiliations:** ^1^ Department of Biosciences College of Science Swansea University Swansea UK; ^2^ School of Medicine Institute of Life Science Swansea University Swansea UK; ^3^ Department of Environmental Sciences Emory University Atlanta GA USA; ^4^ National Botanic Garden of Wales Llanarthne UK; ^5^ Aberystwyth University Aberystwyth UK; ^6^ Institute of Biological, Environmental and Rural Sciences Aberystwyth University Aberystwyth UK

**Keywords:** DNA metabarcoding, generalisation, grassland, hoverfly, pollination, pollination networks, specialisation

## Abstract

1. Pollination by insects is a key ecosystem service and important to wider ecosystem function. Most species‐level pollination networks studied have a generalised structure, with plants having several potential pollinators, and pollinators in turn visiting a number of different plant species. This is in apparent contrast to a plant's need for efficient conspecific pollen transfer.
2. The aim of this study was to investigate the structure of pollen transport networks at three levels of biological hierarchy: community, species and individual. We did this using hoverflies in the genus *Eristalis*, a key group of non‐Hymenopteran pollinators.
3. We constructed pollen transport networks using DNA metabarcoding to identify pollen. We captured hoverflies in conservation grasslands in west Wales, UK, removed external pollen loads, sequenced the pollen DNA on the Illumina MiSeq platform using the standard plant barcode *rbcL*, and matched sequences using a pre‐existing plant DNA barcode reference library.
4. We found that *Eristalis* hoverflies transport pollen from 65 plant taxa, more than previously appreciated. Networks were generalised at the site and species level, suggesting some degree of functional redundancy, and were more generalised in late summer compared to early summer. In contrast, pollen transport at the individual level showed some degree of specialisation. Hoverflies defined as “single‐plant visitors” varied from 40% of those captured in early summer to 24% in late summer. Individual hoverflies became more generalised in late summer, possibly in response to an increase in floral resources. *Rubus fruticosus* agg. and *Succisa pratensis* were key plant species for hoverflies at our sites
5. Our results contribute to resolving the apparent paradox of how generalised pollinator networks can provide efficient pollination to plant species. Generalised hoverfly pollen transport networks may result from a varied range of short‐term specialised feeding bouts by individual insects. The generalisation and functional redundancy of *Eristalis* pollen transport networks may increase the stability of the pollination service they deliver.

## 1 INTRODUCTION

The structure and function of pollination networks have been the subject of considerable research interest (Jordano, 2016; Nicolson & Wright, 2017; Petanidou, Kallimanis, Tzanopoulos, Sgardelis, & Pantis, 2008). Despite examples of remarkable mutualisms between specific plants and their pollinator species (Johnson, Hollens, & Kuhlmann, 2012; Stokl, Brodmann, Dafni, Ayasse, & Hansson, 2011), plant–pollinator networks often have a generalised structure (Bascompte, Jordano, Melian, & Olesen, 2003; Memmott, 1999; Waser, Chittka, Price, Williams, & Ollerton, 1996) in which plant species are visited by numerous pollinator taxa, and pollinators in turn visit a number of plant species. Pollination is a key ecosystem service (IPBES 2016) that has significant economic value as well as facilitating wider ecosystem function (Gill et al., 2016; Potts et al., 2016). Understanding the structure of plant–pollinator networks is important to safeguarding the provision of this ecosystem service, because generalised networks may be more robust to a number of environmental stressors, including climate change (Gilman, Fabina, Abbott, & Rafferty, 2012; Memmott, Craze, Waser, & Price, 2007), species extinctions and invasive species (Kaiser‐Bunbury, Muff, Memmott, Müller, & Caflisch, 2010; Memmott, Waser, & Price, 2004), and to the impact of habitat management (Vanbergen et al., 2014).

However, generalised pollination networks appear to be contrary to the need of plants for efficient conspecific pollen transfer to achieve pollination (Waser, 1986). It has been suggested that such networks can be both generalised and specialised at different levels of biological hierarchy, with individual insects engaging in short‐term specialised feeding bouts, and therefore efficiently moving pollen between plant conspecifics, whilst networks at the species and community level remain generalised (Armbruster, 2016; Brosi, 2016; Ollerton, 1996). Addressing this issue requires the investigation of individual pollinator behaviour, but is constrained by the limitations of existing techniques, such as following insects in the field (Ambrosino, Luna, Jepson, & Wratten, 2006; Brosi & Briggs, 2013), or morphologically identifying pollen grains carried by insects (Golding & Edmunds, 2003). In particular, the accurate visual identification of pollen requires considerable skill (Bruni et al., 2015; Hawkins et al., 2015) with some plant species groups being difficult to distinguish, even by experts (Galimberti et al., 2014).

Many studies of pollination networks have focussed on bees (Hymenoptera) (Ballantyne, Baldock, & Willmer, 2015; Tucker & Rehan, 2016). However, hoverflies (Syrphidae), which, as adults, feed almost exclusively on nectar and pollen, are also pollinators of a wide range of plants (Gyan & Woodell, 1987; Woodcock, Larson, Kevan, Inouye, & Lunau, 2014), including crop species such as oilseed rape *Brassica napa* (Stanley, Gunning, & Stout, 2013). Wild pollinators, including hoverflies, have been shown to be more effective pollinators (in terms of fruit set) than honeybees in a range of crop systems (Garibaldi et al., 2013), and the species diversity of wild pollinators may make them more resilient to temporal environmental change than managed honeybees (Rader et al., 2015). Nonetheless, there remain key gaps in the pollination science evidence base, particularly relating to which insects pollinate which wild plants (Dicks et al., 2013).

DNA metabarcoding, the use of next‐generation DNA sequencing to identify species from mixed samples (Creer et al., 2016), has great potential in the study of insect pollen transport (Clare, Schiestl, Leitch, & Chittka, 2013). This approach compares samples of mixed DNA sequences recovered from pollen with a library of plant species sequences (Hawkins et al., 2015). DNA barcodes have been successfully recovered from pollen carried by bees (Bell, Loeffler, & Brosi, 2017; Bell, Fowler, et al., 2017; de Vere et al., 2017; Wilson, Sidhu, LeVan, & Holway, 2010). DNA metabarcoding therefore has the potential to offer an insight into pollen transport by hoverflies, by allowing the identification of mixed pollen samples from individual hoverflies without requiring specialist palynological expertise (Bell et al., 2016). Such pollen transport networks can give an insight into hoverfly foraging behaviour, and thus which plants are of importance as food resources, which is critical to their conservation (Fowler, Rotheray, & Goulson, 2016; Milberg et al., 2016; Pontin, Wade, Kehrli, & Wratten, 2006). It can also give some indication of their role in pollination (Jauker, Bondarenko, Becker, & Steffan‐Dewenter, 2012; Nicolson & Wright, 2017).

Here, we investigate the pollen transport network of *Eristalis* hoverflies, a genus widely distributed across the Holarctic. We carried out this study in fen‐meadow grasslands, a floristically rich habitat of conservation importance in lowland Wales, UK (Blackstock, Howe, & Stevens, 2010). We retrieved and isolated pollen DNA carried on the bodies of hoverfly specimens, and sequenced and matched sequences to a pre‐existing library to identify the pollen plant taxa (de Vere et al., 2012). We also quantified the flower resource available to hoverflies in these botanically diverse habitats. From these data, we constructed hoverfly pollen transport networks that describe specialisation at the level of the overall network (*H*_2_ *′*) and species (*d*′) level (Bluthgen, Menzel, & Bluthgen, 2006). Using the relative proportions of pollen sequence reads, we have investigated the degree of specialisation shown by individual insects. This has allowed us to investigate hoverfly pollen transport from whole networks to individuals and relate these results to changes in flower resource availability. We address the following specific research questions:

1. What plant pollens are *Eristalis* hoverflies transporting, and how do the proportions of different pollen species change during the summer flight period? We predict that hoverflies carry pollen reflecting seasonal variation in floral resource availability, and become less specialised later in the season as more pollen resources became available.
2. How are *Eristalis* pollen transport networks structured? Our prediction is that, similar to pollination networks studied previously, they would have a generalised structure at the whole network and species level.
3. Are individual insects specialised? Our prediction here is that, given the evidence of floral constancy found by direct observation of hoverflies during foraging bouts (Goulson & Wright, 1998), some degree of specialisation will be reflected in the pollen loads of individual insects.

## 2 MATERIALS AND METHODS

### 2.1 Field collection of hoverflies

The study took place during 2014 at four grassland sites of high conservation importance in west Wales, United Kingdom. We collected *Eristalis* hoverflies at these locations (referred to as “CAD,” “LLC,” “RHC” and “TRE”), where the National Vegetation Classification community *Molinia caerulea—Cirsium dissectum* fen‐meadow (*Cirsio—Molinietum caerulae)* (Rodwell et al., 1991) was present (for full site information, see Appendix S1) Each site consisted of a single field, surrounded by hedgerows. Collection occurred in two time periods: “early” (1 June–15 July) and “late” (16 July–31 August), between 11:00 and 15:00. To ensure the insects captured were representative of the site as a whole, a series of transects 20 m apart were walked across each site, during which *Eristalis* hoverflies were actively collected using a hand‐held net. Transects were walked continuously, repeating them as necessary, with each site searched for approximately 3 hr in each time period (early and late season). Sites were searched for a single day, but in some instances, were searched over two days, for a total of 3 hr, when poor weather conditions reduced insect activity. Collection dates between the early and late time periods for a given site were separated by a minimum of 26 days. Insects were stored individually in sterile 1.5‐ml tubes at −20°C prior to pollen removal.

### 2.2 Recording of plant species richness and herb flower resource

We used existing grassland survey information in Bevan, Motley, Stevens, and Bosanquet (2006), together with records of species present in the hedgerows, to create a list of plant species (and therefore a measure of plant species richness) for each site.

To measure the grassland herb flower resource (here termed “flower unit score”), we placed a 50 m × 50 m plot approximately centrally in each site. This size was selected as the largest plot size that could be used on the smallest site. Within the plot, we set up 30 randomly located 1‐m^2^ quadrats, within which we recorded all the plant species in flower, excluding grasses and sedges. We also recorded the number of floral units within the quadrat. For most plant species, a floral unit corresponds to a single flower, but for Apiaceae species, an inflorescence was counted as one flower unit, and for *Dactylorhiza* spp., *Narthecium ossifragum* and *Calluna vulgaris*, a single flowering stem or spike was counted as one flower unit. These measurements are similar to the “blossom units” described by Dicks, Corbet, and Pywell (2002), based on a floral unit that a medium‐sized bee has to fly, rather than walk, between. We recorded the flowers twice at each site, once in the early period, and once in the late.

### 2.3 Pollen removal

We removed external pollen from insects, first via an initial washing of insects in the tube in which the insect had been placed in the field. The fly was immersed in 1 ml of a 1% sodium dodecyl sulphate (SDS) and 2% polyvinyl pyrrolidinone (PVP) solution in water. The tube was shaken vigorously by hand for 1 min and then centrifuged briefly to ensure that the insect was fully immersed in the liquid. It was then allowed to stand at room temperature for 5 min. The tube was then shaken vigorously by hand for 20 s. The fly was removed using forceps to a clean 1.5‐ml tube and frozen at −20°C for subsequent species identification. The tube containing the detergent and pollen was centrifuged at 13,000 rpm for 5 min.

### 2.4 DNA extraction

We used the DNeasy plant mini kit (Qiagen) to extract DNA. The supernatant was discarded and the pellet suspended in 400 μl AP1 and 80 μl proteinase K (1 mg/ml). This was incubated for 60 min at 65 °C in a water bath and then disrupted using a TissueLyser II (Qiagen) for 4 min at 30 Hz with 3‐mm tungsten carbide beads. The subsequent steps were followed according to the manufacturer's instructions, with the exception that QIAshredder column and second wash stage were omitted.

### 2.5 Amplification and sequencing: Illumina Miseq

We amplified the DNA using the *rbcL* DNA barcode marker region (Bell, Loeffler, et al., 2017; CBOL Plant Working Group et al. 2009). Two rounds of PCR were carried out: a primary tailed amplification of the *rbcL* region, followed by a second round of amplification that added the Illumina Nextera index adaptor sequences so that samples could be processed on Illumina platforms and be subsequently separated via bioinformatic processing. We initially amplified the samples using the universal primers *rbcLaf* and *rbcLr506* (de Vere et al., 2012), to which adaptor 5′ overhangs had been added:

*rbcLaf*+adaptor: TCGTCGGCAGCGTCAGATGTGTATAAGAGACAGATGTCACCACAAACAGAGACTAAAGC
*rbcLr506 *+* *adaptor: GTCTCGTGGGCTCGGAGATGTGTATAAGAGACAGAGGGGACGACCATACTTGTTCA.

We performed the PCR using a final volume of 20 μl. A mix of 10 μl of 2× Phusion Mastermix (New England Biolabs), 0.4 μl of 5 μM forward primer (*rbcLaF*+adaptor), 0.4 μl of 5 μM reverse primer (*rbcLr506 *+* *adaptor) and 7.2 μl of molecular biology grade water was made, to which 2.0 μl of template DNA was added. The PCR conditions were as follows: 95°C for 2 min; 95°C for 30 s, 50°C for 1 min 30 s, 72°C for 40 s (35 cycles); 72°C for 5 min, 30°C for 10 s. PCR products were visualised using agarose gel electrophoresis to confirm successful amplification.

We purified the products from the first PCR following IIlumina's 16S Metagenomic Sequencing Library Preparation protocol (Illumina 2013) using Agencourt AMPure XP beads (Beckman Coulter). The Index PCR stage (following the Illumina protocol) used a 25 μl reaction (12.5 μl of 2× Phusion Mastermix, 2.5 μl of Nextera XT i7 Index Primer, 2.5 μl of Nextera XT i5 Index Primer, 5 μl of PCR‐grade water and 2.5 μl of purified first‐round PCR product). PCR clean‐up 2 of the Illumina protocol was then followed, cleaning 20 μl of Indexed PCR product, with a 1:0.8 ratio of product to AMPure XP beads.

We quantified the amplified products using a Qubit fluorescence spectrophotometer (Life Technologies) and pooled at equal concentrations to produce the final library. This was again quantified via Qubit to determine concentration and adjusted to 10 nM concentration with 0.1 M Tris‐HCl/0.01% Tween‐20 solution prior to sequencing on an Illumina MiSeq platform. Library denaturation and sample loading steps followed the Illumina protocol: sample was loaded at 3pM concentration with 20% PhiX control spike and paired‐end sequences generated in 2 × 300 bp format.

### 2.6 Data analysis

A data analysis pipeline was created to process the Illumina sequence reads and to match them to known taxa within a local reference database. Files containing the sequence reads used in this study are available through the NCBI sequence read archive (SRA accession SRP076527). The source code and tools used for the pipeline are available on GitHub at https://github.com/colford/nbgw-plant-illumina-pipeline. Sequences were quality trimmed and then merged. Only sequences greater than 450 bp were used in downstream analysis.

A local blast database was created from *rbcL* sequence data. This includes reference data for all UK native species (de Vere et al., 2012) together with sequences from GenBank for non‐native species known to be found in the UK. Using this database allowed for unexpected identifications, particularly of non‐native species. Each sequence was compared against this database using megablast, and the top 20 maximum bit scores were returned. If these scores matched to a single species, the sequences were assigned to that species. If 60% or more of the sequences matched to a single genus, the sequences were assigned to that genus. blast results that did not fall into these two categories were assigned to the category “various.”

All results were then checked and verified using expert knowledge. This integrates knowledge of local habitats, species distribution and rarity to support the blast identifications to species and genus and to identify sequences assigned as “various” to family or tribe level where possible. Any remaining sequences blasting to multiple families were classified as “unknown” (Hawkins et al., 2015; de Vere et al., 2017).

Results for each pollen sample were manually filtered so that only species recorded within the UK (Stace, 2010) were retained. The number of sequences for each insect was then converted to a proportion (%), to control for differences in DNA amplification between samples in the initial PCR. These results can be influenced by differences in the amount of pollen produced by different plants and biases introduced during DNA extraction, PCR and sequencing. To avoid these biases, pollen results were used on a presence/absence basis for the network analysis, with the percentage data used as a semi‐quantitative measure of DNA amount to investigate the proportions of pollen carried by individuals.

### 2.7 Network analysis

Interaction networks were analysed using the bipartite Package (v. 2.05) (Dormann, Gruber, & Frund, 2008) and binomial‐errors mixed‐effects models using the lme4 package (Bates, Mächler, Bolker, & Walker, 2015) in R version 3.0.1 (R Core Team 2014),

We calculated two metrics of network specialisation (Baldock et al., 2015; Ballantyne et al., 2015; Bluthgen et al., 2006). These were network specialisation (*H*_2_ *′*), which represents the overall level of specialisation of all species in a network, and varies from 0 (complete generalisation) to 1 (complete specialisation); and *d*′, which measures how exclusive a given species' interactions are compared to the other species in a network, and varies from 0 (no exclusivity) to 1 (completely exclusive).

To investigate the influence of time period and hoverfly sex on individual specialisation, we used a binomial‐errors mixed‐effects model. Hoverflies were placed in two categories: predominantly “single‐plant visitors,” defined as individuals where the proportion of sequences of the greatest pollen taxon was 90% or above, and “multiplant visitors,” where the proportion was below 90%. The threshold of 90% was selected because of the possibility of hoverflies acquiring heterospecific pollen from a plant deposited by a previous visitor, or windblown pollen present in the wider environment. It was therefore unrealistic to expect 100% of pollen carried by a hoverfly to come from one plant taxon group. The response variable was single versus multiplant visitors, with time period and hoverfly sex as fixed effects. Site and species were included as random effects.

## 3 RESULTS

### 3.1 Overview

Pollen sequences from Illumina MiSeq were recovered from 180 out of 192 individual hoverfly samples (55 during the early period and 125 in the late period). A total of 2,349,247 (148,216 from early period, and 2,201,031 from late period) sequences over 450 bp in length could be attributed to tagged sequences of *rbcL*. Of these, 2,330,020 (99.2%) could be identified to plants at species, genus or family level (see Appendix S2). A total of 65 plant taxonomic groups were identified consisting of 24 species, 27 genera and 14 tribes and families, ranging from 31 at site TRE to 39 at site LLC. Hoverflies were identified to six species: *E. arbustorum* (*n* = 5), *E. horticola* (*n* = 57), *E. intricaria* (*n* = 2), *E. nemorum* (*n* = 41), *E. pertinax* (*n* = 53) and *E. tenax* (*n* = 17).

### 3.2 What plant pollens are *Eristalis* hoverflies transporting?

Botanical surveys of sample sites showed that plant species richness varied from 63 (site CAD) to 83 species (site RHC). Flower unit score, recorded twice at each site, ranged from 20 floral units (site LLC early period) to 631 floral units (site RHC early period) (Table 1). Flower unit scores rose at three of four sites from the early to the late period. Flower unit scores fell at one site (RHC), although it should be noted that resources at this site in early summer were exceptionally high relative to the other sites, and the value in late summer was comparable to sites LLC and TRE.

**Table 1:** The total number of plant taxonomic groups recorded from pollen carried by *Eristalis* hoverflies at four sites (“CAD,” “LLC,” “RHC” and “TRE”) in west Wales during 2014, with site plant species richness and flower unit score (see text for definition) between 1 June and 15 July (early) and 16 July and 31 August (late)

Site	CAD	LLC	RHC	TRE
No. Pollen taxa recorded	32	39	38	31
Site plant species richness	63	75	83	66
Flower unit score Early	168	20	631	75
Flower unit score Late	372	100	96	99

**Figure 1:**
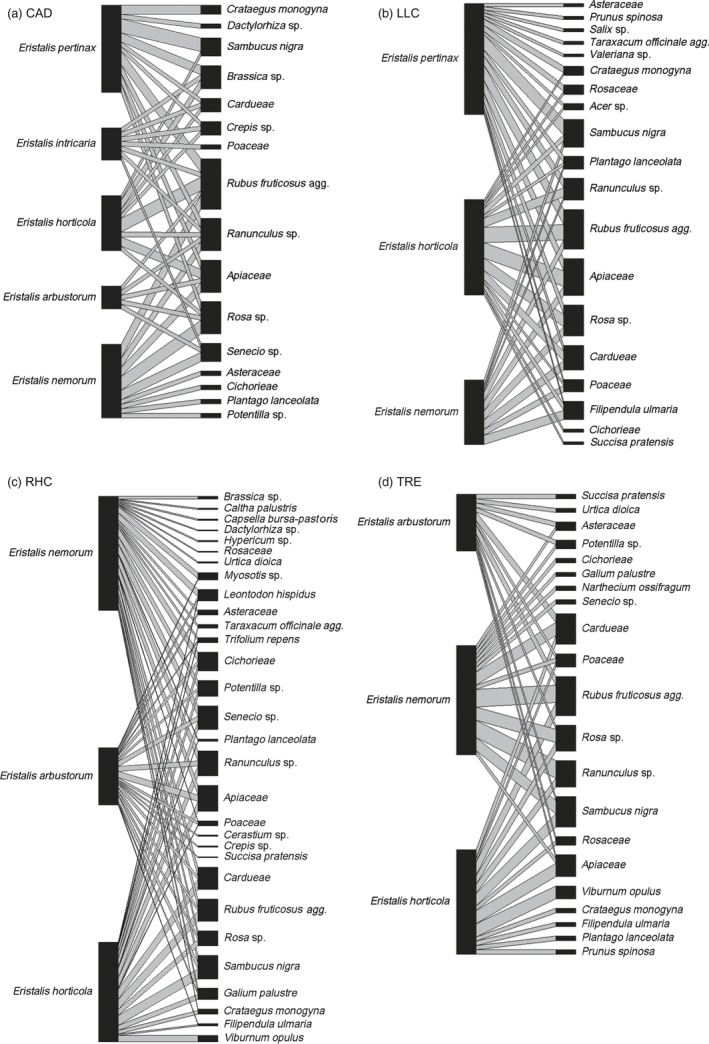
*Eristalis* hoverfly pollen transport networks at four grassland sites CAD (top left), LLC (top right), RHC (bottom left) and TRE (bottom right). Insects collected between 1 June 2014 and 15 July 2014 (“early”)

**Figure 2:**
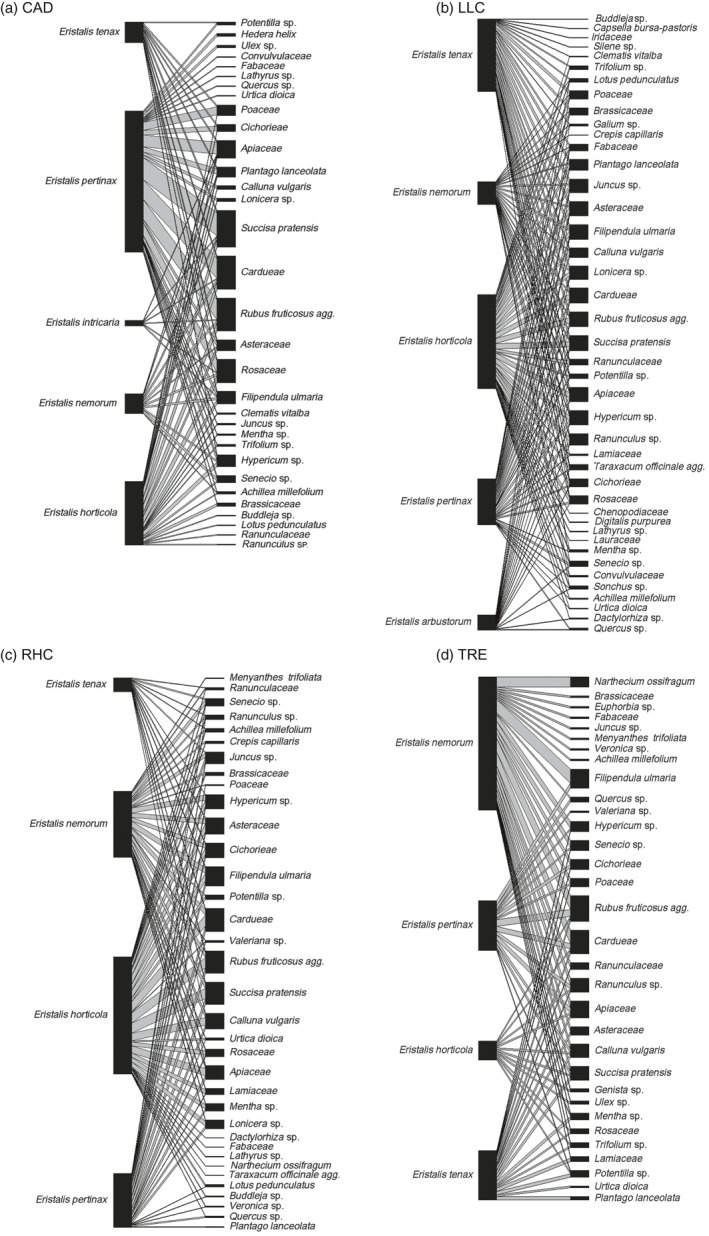
*Eristalis* hoverfly pollen transport networks at four grassland sites CAD (top left), LLC (top right), RHC (bottom left) and TRE (bottom right). Insects collected between 16 July 2014 and 31 August 2014 (“late”)

**Figure 3:**
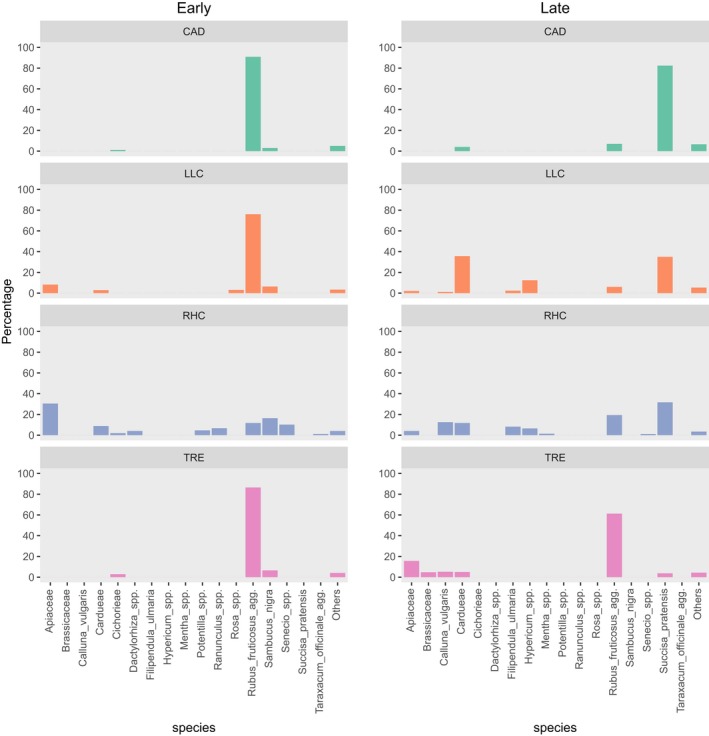
Proportions (%) of pollen DNA sequences from hoverflies on four grasslands. Early—insects collected between 1 June and 15 July. Late—insects collected between 16 July and 31 August. Pollen taxa contributing 1% or less to the total are combined into the “others” category

When all sites were considered together, during the early period the most frequently recovered pollen originated from *Rubus fruticosus* agg., *Sambucus nigra*, Apiaceae, *Ranunculus* spp. and Cardueae (thistles and knapweeds). In the late period, the plant pollens present on most *Eristalis* hoverflies were Cardueae, *R. fruticosus* agg., *Succisa pratensis*,* Filipendula ulmaria* and Apiaceae (see Appendix S2). Whilst the sites CAD, LLC and TRE were similar in the numbers of different pollen taxa present, site RHC was more plant species‐rich and had a greater variety of pollen taxa carried by hoverflies. This was particularly noticeable in the early period, when *R. fruticosus* agg. was the predominant taxon at CAD, LLC and TRE, but at RHC there was a mix of pollens, with Apiaceae, *R. fruticosus* agg., *S. nigra* and *Senecio* spp. being the principal taxa recovered from hoverflies (Figure 1, 2, 3).

### 3.3 How are *Eristalis* pollen transport networks structured?

The numbers of *Eristalis* individuals identified, together with network metrics, *H*_2_ *′* and *d*′, are shown in Table 2. The network specialisation metrics *H*_2_ *′* indicate that networks were generalised, with all values below 0.3 (Bluthgen et al., 2006). *H*_2_ *′* values ranged from 0.071 (LLC late) to 0.298 (TRE early). Values of *H*_2_ *′* fell from the early period to the late period at all sites, indicating that network generalisation increased during the summer.

**Table 2:** Values of network metric *H*′_2_ and the species interaction specialisation metric *d*′ for *Eristalis* hoverflies at four grassland sites in west Wales from 1 June to 15 July (early) and 16 July to 31 August (late) in 2014

	Early								Late								Mean *d*′ all sites (*SD*)
	CAD		LLC		RHC		TRE		CAD		LLC		RHC		TRE		
Network specialisation *H*′_2_	0.279		0.133		0.130		0.298		0.117		0.071		0.079		0.238		
Species specialisation *d*′	*d*′	*n*	*d*′	*n*	*d*′	*n*	*d*′	*n*	*d*′	*n*	*d*′	*n*	*d*′	*n*	*d*′	*n*
*Eristalis arbustorum*	0.02	1		0	0.09	5	0.17	2		0	0.09	2		0		0	0.07 (0.061)
*Eristalis horticola*	0.08	3	0.01	5	0.11	8	0.15	3	0.08	9	0.03	11	0.04	17	0.20	1	0.09 (0.064)
*Eristalis intricaria*	0.24	1		0		0		0	0.00	1		0		0		0	0.12 (0.170)
*Eristalis pertinax*	0.15	3	0.05	6		0		0	0.06	25	0.04	6	0.07	9	0.15	4	0.09 (0.050)
*Eristalis nemorum*	0.32	3	0.11	3	0.08	8	0.15	4	0.09	3	0.04	3	0.05	9	0.14	8	0.12 (0.089)
*Eristalis tenax*		0		0		0		0	0.08	3	0.06	10	0.07	2	0.20	2	0.10 (0.066)

Values of the species level metric *d*′ at all sites were low (Table 2), ranging from 0.00 (*E. intricaria* at site CAD late) to 0.32 (*E. nemorum* at site CAD early). This indicates that very few hoverfly–plant interactions were exclusive to a particular hoverfly species at any site in either time period. When all sites in the early period are considered, *d*′ values ranged from 0.01 to 0.32, whilst in the late period, they ranged from 0.00 to 0.20. The mean value of *d*′ for a species across all sites and time periods varied from 0.07 (*E. arbustorum*) to 0.12 (*E. intricaria* and *E. nemorum*), although the small sample sizes should be noted, particularly in the case of *E. arbustorum* and *E. intricaria*. When the degree of specialisation in a species at the same site between time periods was considered, almost all values of *d*′ fell from early summer to late summer, with the exception of *E. horticola* at site LLC and at TRE.

### 3.4 Are individual hoverflies specialised?

The *Eristalis* species in this study are all morphologically similar honeybee mimics, with the exception of *E. intricaria*, a bumblebee mimic. They are also of similar size, with a thorax width ranging from 3.11 mm (female *E. arbustorum*) to 4.26 mm (female *E. tenax*), and proboscis length ranging from 5.33 mm (male *E. arbustorum*) to 7.28 mm (male *E. tenax*) (F. Gilbert, personal communication). Data were therefore pooled across all species to investigate individual specialisation. Results from the binomial‐errors mixed‐effects model showed that multispecies plant visitors (defined as individuals for which 90% or more of pollen sequences came from a single‐plant taxon) were significantly more common in the late versus the early time period (*z* = 2.712, *p *<* *.01), but that sex was not significantly related to the proportion of multispecies visits.

**Figure 4:**
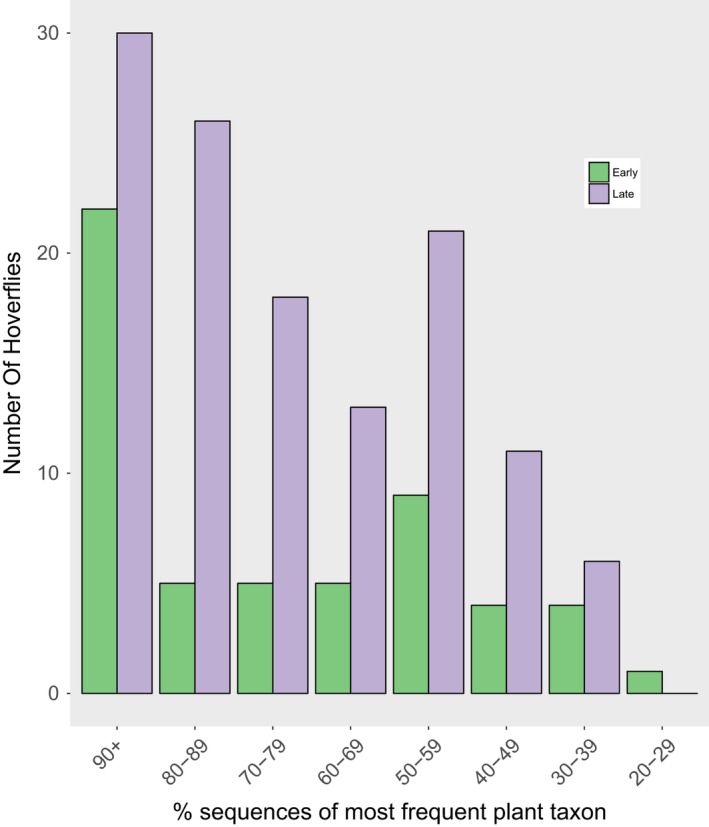
The percentage categories of the most frequent pollen DNA sequences from a single‐plant taxon recovered from individual *Eristalis* hoverflies at four grassland sites in early (*n* = 55) and late (*n* = 125) summer 2014

The proportions of sequences from individual hoverflies arising from a single‐plant taxon are shown in Figure 4. Most of the pollen on hoverfly individuals came from a single‐plant taxon. In the early period 22 of 55 (40%), hoverflies had 90% or more of their pollen sequences coming from a single‐plant taxon, and 37 of 55 (67%) had at least 60% of their pollen sequences from a single‐plant taxon. In the late period, 30 of 125 (24%) had 90% or more of their pollen sequences coming from a single‐plant taxon, and 87 of 125 (70%) had at least 60% of their pollen sequences from a single‐plant taxon.

## 4 DISCUSSION

Our results demonstrate that pollination transport networks amongst *Eristalis* hoverflies are generalised, but that this generalisation may be a consequence of short‐term specialisation by individuals on particular plant species. The results have implications for the effectiveness of hoverflies as potential pollinators, and the role they play in the functioning of the grassland ecosystems used in this study.

All of the networks were generalised, with *H*_2_ *′* values below 0.3. These values are comparable to those recorded in flower–visitor networks in bumblebees (*Bombus*) (Ballantyne et al., 2015) and moths (Lepidoptera) (Banza, Belo, & Evans, 2015). The increasing generalisation (i.e., lower *H*_2_ *′* value) during the summer may reflect the increasing amount and diversity of flower resources as the summer progresses. This is similar to the results of Baldock et al. (2015), who attributed generalisation in mixed pollinator networks in urban areas to the greater diversity of plants, including many non‐native species. Our results are consistent with these and other studies that have described generalised pollen transport systems in temperate systems (Devoto, Bailey, & Memmott, 2011; Forup, Henson, Craze, & Memmott, 2008; Marrero, Torretta, & Medan, 2014).

Specialisation at the species level in our pollen transport networks, as described by *d*′, was extremely low. This demonstrates that there is considerable functional redundancy in pollen transport amongst *Eristalis* species at our sites. Functional redundancy within ecological networks can increase ecosystem service stability (Russo, Debarros, Yang, Shea, & Mortensen, 2013) and robustness to extinctions (Kéfi, Miele, Wieters, Navarrete, & Berlow, 2016). However, the small sample sizes, particularly for *E. arbustorum*,* E. intricaria* and *E. tenax* should be noted. Further work will be required to fully establish any specialisation at the species level amongst *Eristalis* hoverflies. In addition, any functional redundancy may not extend to other habitats, as a species that is functionally redundant in one system may be pivotal in another (Fetzer et al., 2015).

Goulson and Wright (1998) demonstrated floral constancy by two species of hoverfly, *Episyrphus balteatus* and *Syrphus ribesii*. In our study, between 40% (early period) and 24% (late period) of hoverflies had at least 90% of their pollen sequences from a single‐plant taxon, with the majority having at least 60% of sequences from a single‐plant taxon. This suggests that individuals are showing some degree of specialisation. The number of hoverflies appearing to be visiting a single‐plant taxon declined during the summer, possibly as a result of increasing flower resources. Hoverflies can have colour preferences (Sutherland, Sullivan, & Poppy, 1999), which may facilitate constancy, and evidence that hoverflies fly longer distances between feeding bouts than bees has been attributed to them not being central place foragers (Lysenkov, 2009). The presence of predators and variation in feeding resources can also influence foraging behaviour in *Eristalis tenax* (Llandres, De Mas, & Rodriguez‐Girones, 2012). Our results show that flower constancy, as inferred by pollen loads, was a feature of foraging by *Eristalis* species in our study. However, further work is required to describe flower constancy in hoverflies and hoverfly foraging behaviour.

Whilst most plant–pollinator interactions studied appear to be generalised (Bosch, Martín González, Rodrigo, & Navarro, 2009; Ollerton et al., 2009), the limited flower constancy described above may ensure efficient pollination. Generalisation and specialisation can occur simultaneously (Brosi, 2016; Ollerton, 1996), because whilst individual behaviour during a short‐term foraging bout may be specialised, overall pollination by species and communities can be generalised. Our results support this view, with some degree of relatively specialised pollen transport at the individual level, but generalised at the species and network levels. Generalised hoverfly networks may therefore be an emergent property of a diverse set of individual short‐term specialisms. This result is consistent with Tur, Vigalondo, Trøjelsgaard, Olesen, and Traveset (2014), who investigated pollen transport from the whole network to individual insect level using microscopic palynological techniques. The causes of flower constancy are still debated, although it has been argued that such behaviour in social bees is an adaptive strategy to maximise resource use in a varied environment (Grüter & Ratnieks, 2011). How flower constancy in hoverflies arises in hoverflies is unclear, although it has been explained as resource partitioning between species (Haslett, 1989). Further research is required to explore the reasons behind flower choice by individual hoverflies.

Our study has revealed the extent to which hoverflies are transporting pollen in grassland systems. Morris (1998) lists 188 plant species visited by all hoverfly species in southern England. In contrast, this study found 65 distinct pollen taxa on *Eristalis* hoverflies at four sites, of which 33 were also recorded as visited by *Eristalis* species by Morris (1998). This indicates that hoverflies are visiting a wider range of plants than has been previously understood based on observations of flower visitation.

Our data indicate that *Rubus fruticosus* agg. and *Succisa pratensis* are critical plants for the hoverfly genus in our study. *Rubus fruticosus* agg. is a very rewarding nectar‐producing plant for many insects (Baude et al., 2016). Hoverflies have also been recorded as key flower visitors for *S. pratensis* (Kwak, 1993), a plant of conservation interest as the food plant of the endangered butterfly *Euphydryas aurinia* (marsh fritillary) (Wahlberg, Klemetti, & Hanski, 2002). Our research suggests that this plant may also be a critical resource for hoverflies, who in turn may be playing an important role in *S. pratensis* reproduction. Both *R. fruticosus* agg. and *S. pratensis* may represent “keystone species” (Memmott, 1999) in these pollen transport networks and may be facilitating the pollination of other plant species by acting as “magnet species” (Johnson, Peter, Nilsson, & Ågren, 2003).

The pollen accumulated on the body of a hoverfly represents a record of its activity. The residence time of a pollen grain on the body of a hoverfly will determine how long that record represents. Hoverflies engage in regular cleaning, by rubbing their legs across their body, wings and eyes (Holloway, 1976). Gilbert (1985) showed that *Eristalis* species spend between 10% and 13% of their time resting, during which time they perform cleaning behaviour. However, this resting behaviour was mostly concentrated between 08.00 and 10.00, and again between 14.00 and 15.00. The remaining time was devoted to feeding or flight between flowers. Although even relatively brief cleaning bouts could potentially remove pollen, and different pollens will have varying adhesive quality, pollen loads could constitute a record of hoverfly behaviour over a significant proportion of a day's activity (Gyan & Woodell, 1987). Almost all insects carried at least two pollen taxa, suggesting that pollen is retained over a long enough period for the insect to have visited several plant taxa without removing pollen through grooming behaviour. Heterospecific pollen deposited on a plant stigma by previous insect visitors may also be acquired by hoverflies, as well as pollen available in the wider environment (Willmer, Cunnold, & Ballantyne, 2017). Both these could act to increase the number of pollen plant taxa carried by hoverflies and give the appearance of a wider range of plant visitation than is actually the case. Exploring the dynamics of pollen transport by hoverflies is an important subject to fully understand the role of these insects in pollination and requires further research.

Bees are recognised to be the most effective insect pollinators in most systems, including grasslands (Willmer et al., 2017). Nonetheless, non‐bee pollinators can be effective pollinators of both wild and crop plants (Horsburgh, Semple, & Kevan, 2011; Orford, Vaughan, & Memmott, 2015; Rader et al., 2015). Our results suggest that individual *Eristalis* hoverflies show a degree of flower fidelity (Brosi, 2016), a trait recognised as increasing pollination effectiveness (Morales & Traveset, 2008). However, transport of pollen by a flower‐visiting species does not necessarily imply that the species is an effective pollinator (Ballantyne et al., 2015; King, Ballantyne, & Willmer, 2013). Therefore, this study can only indicate the potential role that hoverflies may be playing in pollination services and provides some insight into the foraging behaviour of hoverflies themselves. Further work is required, particularly to provide more data on hoverfly pollen loads early in the flight season. Similarly, this work focusses on one widespread genus of hoverflies. Other hoverfly species may have different foraging strategies (Branquart & Hemptinn, 2000; Haslett, 1989), or may utilise other habitats, and consequently carry different pollen loads. Further work is also needed to reconcile pollen transport and actual pollination effectiveness, particularly in non‐Hymenopteran species.

## 5 CONCLUSIONS

There has been considerable debate about the structures of pollination networks, and the generalised nature of such networks, which appear contrary to the requirement of plants for efficient conspecific transport of pollen. Here, we show the value of DNA metabarcoding in the investigation of plant–pollinator interactions, which can reveal relationships more effectively than visit observations (Pornon et al., 2016). By allowing the systematic investigation of pollination networks from the level of individual insects through to whole communities, our results show how generalised networks can emerge from the short‐term specialisation of individuals, thus reconciling generalised network structures with effective plant pollination. This study presents an example of DNA metabarcoding being used in the investigation of pollination by non‐hymenopteran species and adds to the knowledge base of ecosystem service provision. A future focus on integrating flower visitation, pollen transport and pollination effectiveness will allow a more complete description of the structure and function of pollination networks.

## AUTHORS' CONTRIBUTIONS

A.L. conceived the study, undertook fieldwork, DNA sample preparation, statistical analysis and led writing of the manuscript. O.B. performed statistical analysis and drafted the manuscript. B.J.B. performed statistical analysis and drafted the manuscript. C.R.F. undertook data analysis and figure design. D.W.F. and P.J.N. helped to plan the study and draft the manuscript. C.G. designed the pollen removal protocol and drafted the manuscript. M.H. assisted with development of NGS metabarcoding protocols and creation of the sequencing libraries, performed the NGS runs and assisted with data analysis. N.deV. conceived and designed the experiments, acquired and analysed the data, and drafted the manuscript.

## DATA ACCESSIBILITY

Data available from the Dryad Digital Repository: https://doi.org/10.5061/dryad.p412r16 (Lucas et al., 2018). Files containing the sequence reads used in this study are available through the NCBI sequence read archive (SRA accession SRP076527). The source code and tools used for the pipeline are available on GitHub at https://github.com/colford/nbgw-plant-illumina-pipeline, https://doi.org/10.5281/zenodo.1201548 (Ford, 2018).
